# Remote ischemic per-conditioning did not modulate kidney Klotho expression in acute kidney injury induced by renal ischemia/reperfusion injury

**DOI:** 10.1186/s12882-025-04572-8

**Published:** 2025-11-14

**Authors:** Afsoon Afshari, Negar Azarpira, Zeinab Karimi

**Affiliations:** 1 Nephro-Urology Research Center, Shiraz University of Medical Sciences, Shiraz, Iran; 2 Transplant Research Center, Shiraz University of Medical Sciences, Shiraz, Iran

**Keywords:** Klotho, Renal ischemic reperfusion injury, Remote ischemic per conditioning, Acute kidney injury, Inflammation, Oxidative stress

## Abstract

**Background:**

Renal ischemia-reperfusion injury (I/RI) is a major medical problem related to high mortality and morbidity. Klotho plays a critical role in the kidney pathogenesis of I/RI. The current study aimed to investigate the effect of cyclic remote ischemic perconditioning (RIPerC) on renal downregulation of the Klotho protein in bilateral ischemic reperfusion (BIR).

**Material and method:**

Twenty-four Sprague-Dawley rats were divided into (I) sham group which was subjected to abdominal mid-line incision without ischemia; (II) BIR group which was exposed to 60 min ischemia followed by 24 h of reperfusion; and (III) The BIR + RIPerC group which was subjected to the same renal BIR and occlusion of the left femoral artery (cyclic 4*5’/5’). After 24-h, the blood and kidney samples were collected. Plasma creatinine (Cr) levels and blood urea nitrogen (BUN) were determined. Total antioxidant capacity (TAC); total oxidant status (TOS); oxidative stress index (OSI); mRNA levels of IL-6, TNF-α, NF-kβ, IL-10, and klotho; and pathological changes were evaluated in the renal tissues.

**Results:**

BIR resulted in renal dysfunction, as confirmed by higher plasma levels of Cr and BUN and structural changes. This was accompanied by increased TOS levels, OSI index, and decreased TAC levels. IL-6, TNF-α and NF-kβ upregulated, and klotho and IL-10 downregulated after renal ischemia. In the BIR + RIPerC group, RIPerC attenuated the destructive effects of BIR. RIPerC was effective in decreasing oxidative stress and inflammation. However, this procedure cannot upregulate the Klotho gene.

**Conclusion:**

Remote ischemic per-conditioning provides protection against renal ischemic reperfusion injury without the klotho pathway.

## Introduction

Renal ischemia-reperfusion injury (I/RI) is a major reason for acute kidney injury (AKI) that occurs due to renal transplantation, trauma, sepsis, and some vascular surgery [1]. Although various studies have been performed to understand the causes and molecular mechanisms of renal I/RI, it remains a major global public problem. Many kinds of research have demonstrated that inflammation and oxidative stress are involved in the pathophysiology of damages induced by renal I/RI [2, 3]. Inflammatory responses are triggered by the secretion of inflammatory molecules, including IL-1β, IL-6, TNF-α, and CXCL1, through immune and non-immune cells [4]. In addition, in both renal ischemia and reperfusion period, excessive reactive oxygen species (ROS) are released from the injured cells. Accumulation of ROS is also due to decreased activity of enzymatic and non-enzymatic antioxidants, which induce a state of oxidative stress [5].

Remote ischemic conditioning (RIC), that briefly is episodes of non-lethal ischemia which are applied to tissues remote from the target organ, is an endogenous defense that provides protection against ischemic injury [6]. Short-time cyclic RIC can take place during the onset of prolonged ischemia, which is named remote ischemic per-conditioning (RIPerC) [7]. Investigation of the mechanisms involved in RIPerC is important for not only understanding the pathophysiology of its effect but also improving therapeutic lines pointed at mimicking the protective mechanisms in pharmacological preparation.

Several studies have reported the renoprotective effect of RIPerC and proposed that some mediators should be involved in the protective effects of this procedure [7, 8]. They found that the protection induced by RIPerC might result in reduced proinflammatory cytokines and increased total anti-oxidative capacity [9, 10].

On the other hand, klotho was initially recognized as an aging suppressor protein expressed in various tissues and organs, but, by far, its primary source is in the renal tissue [11, 12]. Klotho has extreme biologic actions, such as the ability to attenuate oxidative stress [13], relieve inflammation [14], promote angiogenesis and vascularization [15], and suppress apoptosis and fibrosis [16]. More experimental and human studies have confirmed that Klotho levels are reduced in the kidneys, blood, and urine following acute kidney injury and renal ischemia-reperfusion injury [12, 17]. Hu et al. reported that the administration of soluble Klotho improved ischemic complications in post-ischemic kidneys [18]. Interestingly, klotho is an early functional biomarker of AKI and is down-regulated during renal I/RI [19]. As briefly mentioned above, more recent studies separately reported that the administration of Klotho supplements or the application of pre or post ischemic conditioning along with ischemia induction can each improve acute kidney injury caused by ischemia. This improvement occurs through the supression of inflammation and oxidative stress. Therefore, the aim of the present study is to determine whether cyclic remote ischemic perconditioning can alter the expression or plasma levels of Klotho.

## Materials and methods

All reagents used in this study were commercially obtained and validated for experimental use. RRIDs were retrieved from the SciCrunch RRID Portal where available. For reagents without assigned RRIDs (YTzol Trizol reagent, Addbio SYBR Green Master Mix, AddScript cDNA Synthesis Kit, and KiaZist TAC/TOS kits), we have provided detailed product information and catalog numbers to ensure reproducibility.

All primers used in this study were custom-designed and synthesized by Pishgam- Tehran. Although RRIDs were not available for these specific sequences, we have provided the full primer sequences in Table 1 to ensure reproducibility. Where applicable, similar commercial primer sets are available through vendors such as OriGene and Qiagen, but these do not carry RRID registration.

**Table 1:** Conditions for real-time PCR reaction of studied genes

NCBI Reference Sequence mRNA (Gene ID)	Primer Sequences (5’-3’)	PCR Product Length (bp)^*^	Thermocycling Program	PCR Mix
TNF-α (59086)	F- AACACACGAGACGCTGAAGT R- TCCAGTGAGTTCCGAAAGCC	93	95 °C/10 min, 40 cycles at 95 °C/15 sec, 60 °C/35 sec	SYBR green Master Mix (10 µl;2x concentration), primers: 0.8 µl/10 p mole, Nuclease free Deps Water: 6.4 µl, Template cDNA: 2 µl
NF-κβ (309165)	F-CATACGCTGACCCTAGCCTG R-TTTCTTCAATCCGGTGGCGA	135		
IL-10 (25325)	F-CATGCTCCGAGAGCTGAGG R-AGGCTTGGCAACCCAAGTAA	131		
Klotho (83504)	F-CTGGTTGCCCACAACCTACT R-GGGAACCTAAGGCGATGGAC	106		
IL-6 (24498)	F- CACTTCACAAGTCGGAGGCT R- TCTGACAGTGCATCATCGCT	114		
GAPDH (24383)	F- AGTGCCAGCCTCGTCTCATA R- GAGAAGGCAGCCCTGGTAAC	91		

### Animal model

Male Sprague–Dawley rats (250–260 g) were bought from the Comparative and Experimental Medicine Center of Shiraz University of Medical Sciences. All rats were kept in separate cages with a 12-hour light/dark artificial cycle, at a stable ambient temperature of 23–25 °C, with ad libitum access to water and a standard diet. The animal care and experimental procedures followed the guidelines accepted by the ethics code of IR.SUMS.REC.1400.364, Shiraz University of Medical Sciences.

### Surgery and experimental design

We used a surgical procedure described in our previous research [20]. In the base of Charan et al. [21] the sample size 8 calculated for this study. Assuming a large standardized effect size (Cohen’s d ≈ 1.5), two-sided α = 0.05, and 80% power for the I/R vs. I/R + RIPerC comparison, the required sample size was *n* = 8 rats per group by the standard Z-approximation and cross-checked in G*Power. This approach performed current guidance for animal studies. Rats were randomly assigned (1:1:1) to Sham, BIR, and BIR + RIPerC using a computer-generated list with block size 3, implemented via sequentially numbered, opaque, sealed envelopes by a researcher not involved in surgeries or outcome assessments. Animals from the same cage were distributed across groups to mitigate cage effects. Histopathology scoring was performed under blinded conditions.

All animals were anesthetized intraperitoneally with Ketamine (50 mg/kg; Woerden, Netherlands) and xylazine (10 mg/kg; Alfazyne, Woerden, Netherlands) in the animal cage. The bilateral renal pedicles were clamped for 60 min with a microaneurysm vascular clamp in the bilateral ischemic reperfusion (BIR) group. Based on objective laboratory observations, the pressure applied by the microaneurysm vascular clamp effectively stops blood flow without damaging the vascular surface. A similar surgical procedure was done in the sham group without the clamp of the renal pedicles. For remote ischemic per conditioning (RIPerC) induction, the skin over the left femoral region was incised, and the surrounding connective tissue was dissected to expose the femoral vessels. The femoral artery and vein were carefully separated. The rats then underwent four cycles of left femoral artery occlusion (5 min of ischemia followed by 5 min of reperfusion) at the onset of renal ischemia [10]. Notably, at the end of each episode of ischemia (5-minute ischemia), pulsatile blood flow was observed in the femoral artery after removing the clamp. After the end of the surgery time, the clamps were removed and confirmed for suitable reperfusion of the blood flow to the ischemic kidney. The abdominal incision was then sutured by 2/0 stitches in 2 layers.

**Fig. 1:**
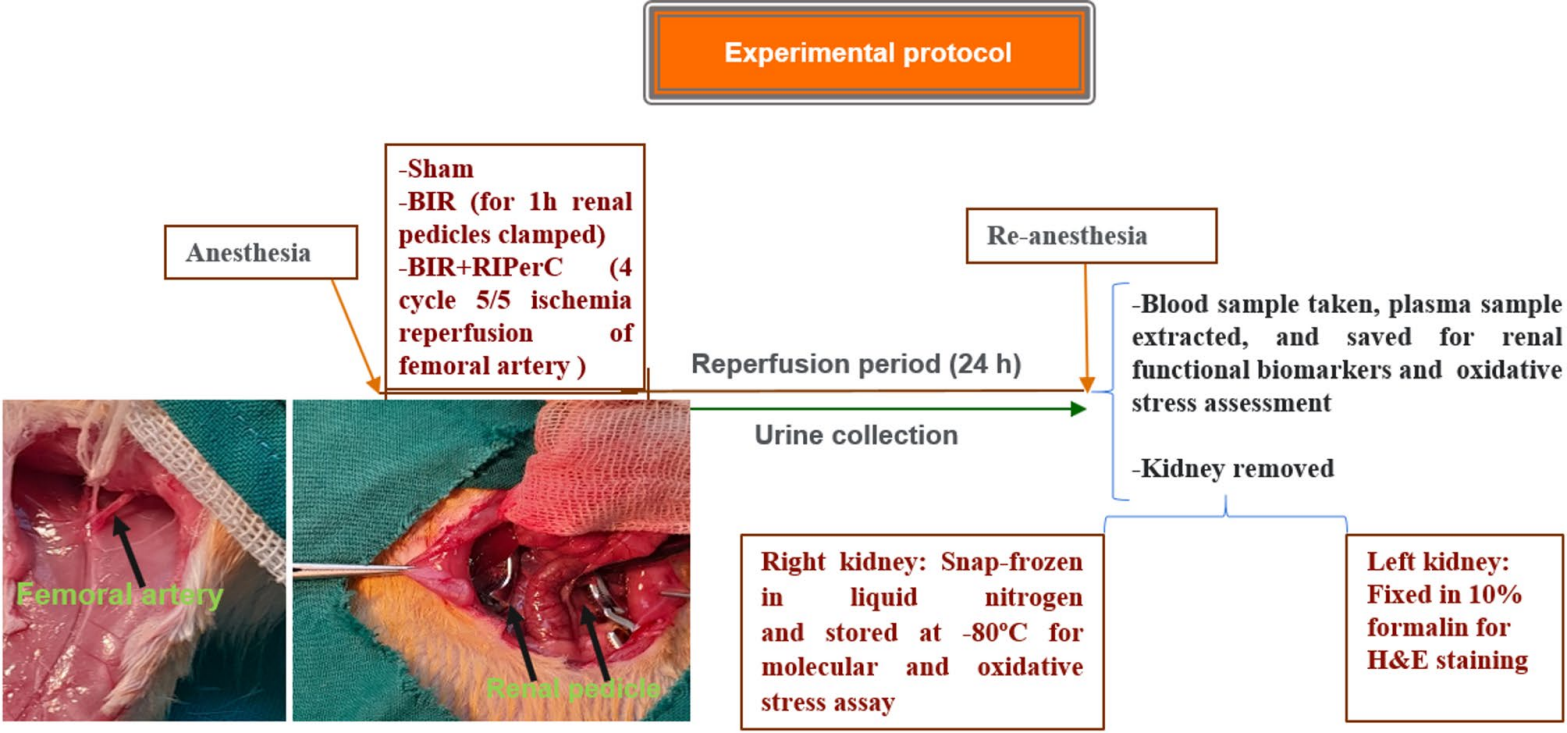
Surgery and experimental design

Twenty-four hours later, all the animals were re-anesthetized and weighed. Blood samples were taken from the tail vein, and the plasma was obtained after centrifugation. Plasma samples were saved at -20 °C to measure the renal functional biomarkers. Subsequently, the kidneys were removed and weighed immediately. The right kidney was frozen at -80 °C for molecular and oxidative stress assay. The left kidney was preserved in 10% neutral buffered formalin for H&E staining (Fig. 1). In this study, we used CO2 gas for the euthanasia of all the rats.

### Biochemical analysis

Plasma creatinine (Cr) and blood urea nitrogen (BUN) were determined using an auto-analyzer (RA-1000 Technicon, America, Namazi Hospital Laboratory, Shiraz, Iran) as renal functional biomarkers.

### Measurement of oxidative stress in the experimental groups

#### Total oxidant status (TOS) assay

The content of total oxidants in the homogenized renal tissues was measured by a commercially available kit (KiaZist, Hamedan, Iran), using a microplate reader (Epoch 2™) at 560 nm wavelength. In brief, oxidants in the sample oxidize the ferrous ion–o-dianisidine complex to the ferric ion. The oxidation reaction was increased by glycerol molecules, which were highly present in the reaction medium. Colored complex was formed by reaction of a ferric ion with xylenol orange in an acidic medium. The total oxidant molecules level of the sample determines the color intensity which could be detected spectrophotometrically (560 nm) [22]. The oxidative assay was standardized using hydrogen peroxide (H_2_O_2_), and the results were reported in terms of nanomolar hydrogen peroxide equivalent per milliliter (nmol H_2_O_2_ Equiv. /ml).

#### Total antioxidant capacity (TAC) assay

The antioxidant capacity of the homogenized renal tissues was measured by a commercially available kit (KiaZist) by a microplate reader (Epoch 2™) at 490 nm wavelength. In this assay, antioxidants present in the sample convert cupric (Cu^2+^) to cupro (Cu^1+^), which produce color when exposed to chromogen solution. The color obtained for test samples was compared with a standard curve obtained with Trolox and reported as nanomoles of Trolox equivalents per milliliter (nmol Trolox Equiv. /mL).

The total protein concentration in the kidney tissues was determined by the bicinchoninic acid protein assay kit (Pierce/Thermo Fisher Scientific, 23227). The final concentration of TOS and TAC for each tissue sample was expressed as nmol/mg protein levels.

#### Oxidative stress index (OSI)

The OSI is an index of oxidative stress which is calculated by the ratio of TOS to TAC using the following formula: OSI (arbitrary unit) = TOS (nmol H_2_O_2_ Equiv./mg of protein) / TAC (nmol Trolox Equiv./mg of protein) × 100 [23].

#### RNA extraction and cDNA synthesis

Each sample (*N* = 24) of kidney tissue from the rats was used to extract total RNA using Trizol (YTzol., Iran). The cDNA synthesis was carried out using the AddScript cDNA Synthesis Kit (Addbio, Korea) following the instructions provided by the manufacturer. The purity and concentration of the total RNA were determined using NanoDrop™ spectrophotometry (Thermo Scientific™, USA) at a wavelength of 260/280 nm.

#### Real-time polymerase chain reaction

The relative expression levels (fold changes, FCs) of studied genes were assessed in kidney tissues of all rats. using SYBR green Real-time PCR. The reactions were performed in duplicate using the Applied Biosystem Step One Plus system with a 96-well optical plate. To ensure accurate normalization, the glyceraldehyde 3-phosphate dehydrogenase (GAPDH) gene was used as the internal control gene. The PCR amplification mix consisted of a PCR master mix containing hot-start Taq DNA Polymerase, SYBR Green I dye, dNTPs mixture, protein stabilizers, and enhancers. Additionally, forward and reverse primers (10 pmol each) and cDNA were included in the mix. The specific sequences of the forward and reverse primers, as well as the real-time PCR conditions, can be found in detail in Table 1.

#### Renal histopathological assessment in the experimental groups

The left kidney was fixed in a 10% formalin solution and embedded in paraffin wax. All histopathological scorings were done on the cortex and medulla area of the renal tissue sections that were cut into 5 μm-thick sections by a microtome and prepared with H&E staining. Tubular and vascular damages were determined with the following criteria: urinary space enlargement, brush border loss, acute tubular necrosis, tubular cast formation, and vascular congestion in 10 fields by non-overlapping and blind fashion (×400 magnification). Renal structural lesions were graded as follows: normal appearance; grade 0, 10–20%; grade 1, 21–40%; grade 2, 41–60%; grade 3, 61–80; grade 4, and 81–100%; grade 5 [24].

### Statistical analysis

Data are expressed as the mean of standard error (SEM). Differences between the groups were evaluated by analysis of variance (ANOVA), followed by Dunnett’s and Tukey’s post-hoc tests. Differences were considered statistically significant at *P* < 0.05, and the most common measure of effect size for a One-Way ANOVA is Eta-squared. Statistical analyses were performed using GraphPad Prism software package ver. 9. (GraphPad Software Inc. La Jolla, California, USA).

## Results

During the surgery, the animal’s body temperature and breathing were carefully controlled, and the procedure was conducted under strictly sterile conditions. Consequently, the mortality rate in the experimental groups was very low—about two rats across the groups.

### Effects of limb ischemic per conditioning on renal biomarkers and histopathological index

**Fig. 2:**
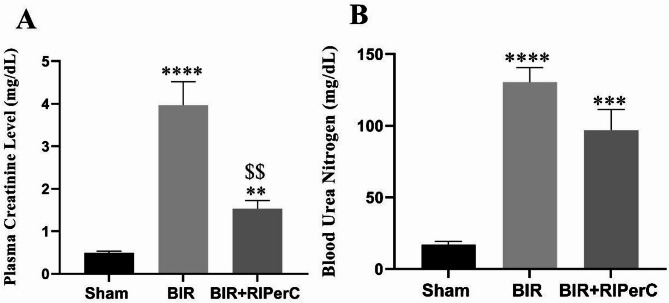
The effect of remote ischemic per-conditioning on the plasma concentration of (**A**) creatinine and (**B**) BUN after 24 h of reperfusion. Statistical analysis was performed using one-way ANOVA, followed by Tukey’s post-test. Data expressed as the mean ± SEM. **p < 0.01, ***p < 0.001 and ****p < 0.0001 compare sham group v.s other groups. ^$$^*p* < 0.01 compare BIR group v.s BIR + RIPerC group. (*n* = 8 rats/group). RIPerC; remote ischemic per-conditioning, BIR; bilateral ischemic reperfusion, BUN; blood urea nitrogen

Renal functional results are shown in Fig. 2. There was a significant difference for BUN and Cr as a renal biomarkers (both *P* < 0.0001, and Eta-squared 0.843 and 0.821 respectively) between groups. Renal functional disturbance were indicated with a significant increase in plasma creatinine and BUN values in the BIR group (3.964 ± 0.55 and 130.4 ± 10.15 mg/dl, respectively) compared with the sham group (0.496 ± 0.04 and 17.0 ± 2.12 mg/dl respectively). RIPerC, along with bilateral renal ischemia significantly decreased the functional biomarker levels in the BIR + RIPerC group. However, the creatinine level in the BIR + RIPerC group was higher than the sham group (0.496 ± 0.04 v.s 1.95 ± 0.25) (Fig. 2A and B).

**Fig. 3:**
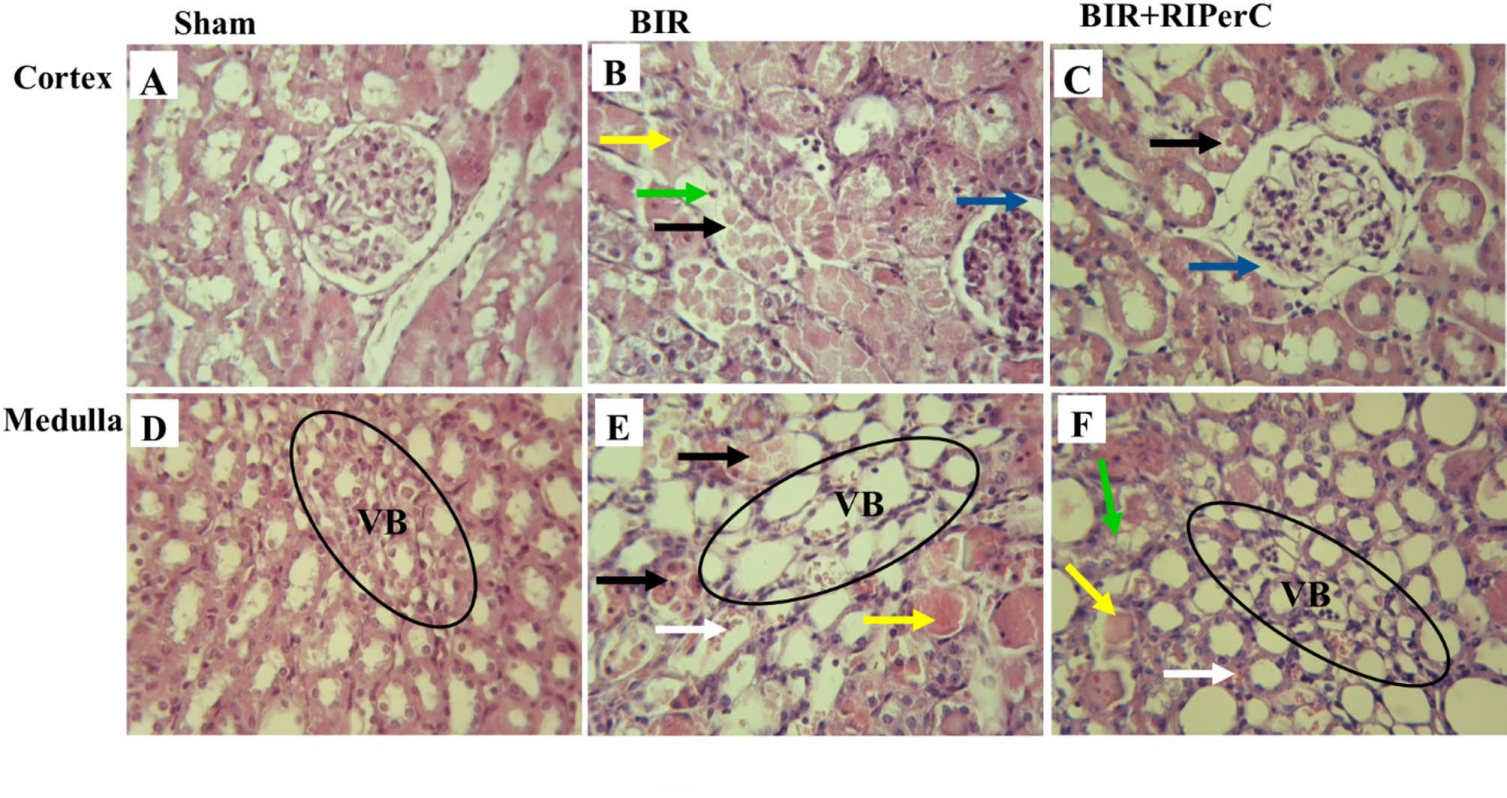
The effect of remote ischemic per-conditioning on histopathological damages of the renal tissue after 24 h of reperfusion. (*n* = 8 rats/group). Exfoliation of the epithelial cells in TAL and PT of the cortex and medulla (black arrows), bowman space enlargement (blue arrows), acute tubular necrosis (green arrow), intratubular cast (yellow arrows), and vascular congestion (white arrows) improved after 24 h of reperfusion in the BIR + RIPerC when compared with the BIR group. RIPerC; remote ischemic per-conditioning, BIR; bilateral ischemic reperfusion, PT; proximal tubule, TAL; tick ascending limb, VB; vascular bundle

Light microscopic examination of the sham-operated group indicated normal appearance in the cortex and medulla area (Fig. 3A and D). In contrast, histopathological evaluation of the renal tissues in the BIR group demonstrated significant acute tubular necrosis and exfoliation of the epithelial cells in the convoluted proximal tubule (PT) at the cortex area (Fig. 3B) as well as in the thick ascending limb (TAL) and pars recta (S3) injury in the medulla area (Fig. 3E). In addition, cast formation and vascular congestion were considerably seen in both areas in the BIR group. Cyclic limb ischemic per conditioning had the ameliorative effect on morphological changes of the renal tissues subjected to 60 min ischemia in the BIR + RIPerC group (Fig. 3C and F).

Quantitative analysis revealed significantly greater tubular and vascular damages in the cortex and medulla compared with the sham group. Remote ischemic conditioning mitigated these pathological changes and reduced injury scores in both the cortex and medulla (Table 2).

**Table 2:** Effects of limb ischemic per conditioning on kidney histopathological criteria

Tubular and vascular damages criteria	Sham	BIR	BIR + RIPerC
**Cortex area**			
Urinary space enlargement	0.00	2.81 ± 0.06 ***	1.60 ± 0.11 **$
Brush border loss	0.00	4.10 ± 0.1 ***	1.50 ± 0.03 ***$$
Acute tubular necrosis	0.00	2.70 ± 0.06 ***	1.10 ± 0.02 ***$
Intratubular proteinaceous cast	0.00	1.50 ± 0.03 **	0.61 ± 0.01 *
**Medulla area**			
Pars recta (S3) injury	0.00	4.50 ± 0.33	2.00 ± 0.16 ***$$
Thick ascending tubule injury	0.00	2.40 ± 0.09 ***	0.73 ± 0.06 ***$$$
Intratubular proteinaceous cast	0.00	3.32 ± 0.41 ***	1.51 ± 0.03 ***$$
Vascular congestion	0.00	3.80 ± 0.11 ***	1.90 ± 0.05 ***$$
**Total histopathological score**	0.00	25.13 ± 1.19	10.95 ± 1.09 ***

Quantitative analysis of tubular and vascular damages criteria in the renal tissue. Data expressed as the mean ± SEM. **p < 0.01and ***p < 0.001 compare groups v.s sham group. ^$^*p* < 0.05, ^$$^*p* < 0.0, and ^$$$^*p* < 0.001 compare BIR + RIPerC with BIR group

### Effects of limb ischemic per conditioning on oxidative stress

**Fig. 4:**
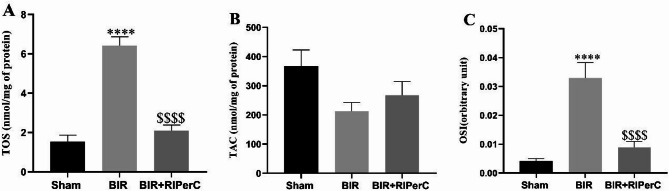
The effect of remote ischemic per-conditioning on the tissue level of TOS (**A**), TAC (**B**), and OSI ratio(**C**) after 24 h of reperfusion. Statistical analysis was performed using one-way ANOVA, followed by Tukey’s post-test. Data expressed as the mean ± SEM. ***p < 0.001 and ****p < 0.0001 compare sham group v.s other groups. *p* < 0.0001 compare BIR group v.s BIR + RIPerC group (*n* = 8 rats/group). RIPerC; remote ischemic per-conditioning, BIR; bilateral ischemic reperfusion, TOS; total oxidant status, TAC; total antioxidant capacity, OSI; oxidative stress index

TOS level and OSI ratio had significant difference among groups (*P* < 0.0001, and Eta-squared 0.895 and 0.7794 respectivley). Renal ischemic reperfusion injury significantly increased the TOS level and OSI (6.41 ± 0.45 and 0.032 ± 0.005, respectively) in the renal tissue sample (Fig. 4A and C). Renal ischemic reperfusion slightly decreased the TAC level (212.3 ± 0.30.09). However, there was not significant difference among groups (*p* = 0.08 and Eta-squared 0.332) for TAC level. (Fig. 4B).

Remote ischemic per conditioning reversed the oxidative stress statues in the BIR + RIPerC group. TOS level and OSI index (2.1 ± 0.28 and 0.008 ± 0.002, respectively) were significantly lower, while the TAC level (267.5 ± 47.36) was higher than the BIR group (Fig. 4A-C).

### Effects of limb ischemic per conditioning on the expression of TNF-α, NF-κβ, IL-6, and IL-10

**Fig. 5:**
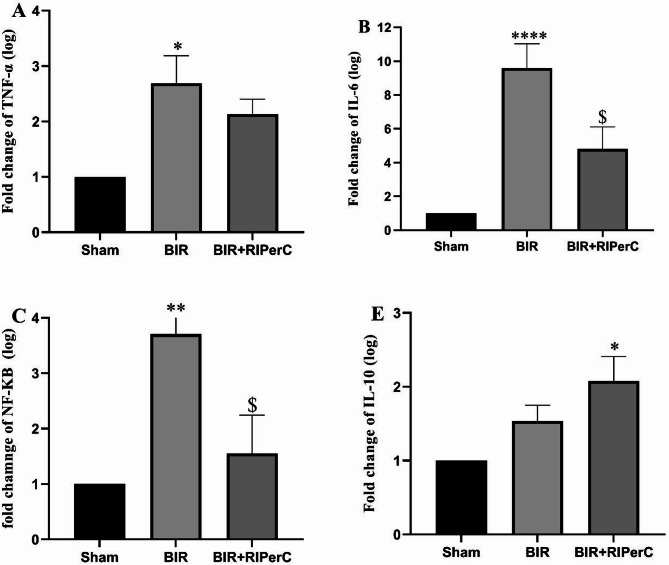
The effect of remote ischemic per-conditioning on the gene expression of TNF-α (**A**), IL-6 (**B**), NF-κβ (**C**), and IL-10 (**D**) after 24 h of reperfusion. Statistical analysis was performed using one-way ANOVA, followed by Tukey’s post-test. Data expressed as the mean ± SEM. *p < 0.05,**p < 0.01, and ****p < 0.0001 compare sham group v.s other groups. ^$^*p* < 0.05 compare BIR group v.s BIR + RIPerC (*n* = 8 rats/group). RIPerC; remote ischemic per-conditioning, BIR; bilateral ischemic reperfusion, I/RI; ischemic reperfusion injury

The expression of TNF-α, NF-Kβ, IL-6, and IL-10 significantly (*P* < 0.05, *P* < 0.001, *P* < 0.0001, and *P* < 0.01, and Eta-squared 0.271, 0.437, 0.713, and 0.346 respectively) changed amonge experimental groups. Sub groups analysis indicated significant up regation of TNF-α, NF-Kβ and IL-6 in the BIR group when compared to the sham group. In addition, the expression of IL-10 was slightly enhanced (Fig. 5E). This is because IL-10 is an anti-inflammatory cytokine that is essential in suppressing the immune response. Remote ischemic per-conditioning relieved inflammation, suppressed the gene expression of TNF-α, NF-Kβ and IL-6, and increased IL-10 in the BIR + RIPerC group (Fig. 5A-D).

### Effects of limb ischemic per conditioning on the mRNA and plasma level of Klotho

**Fig. 6:**
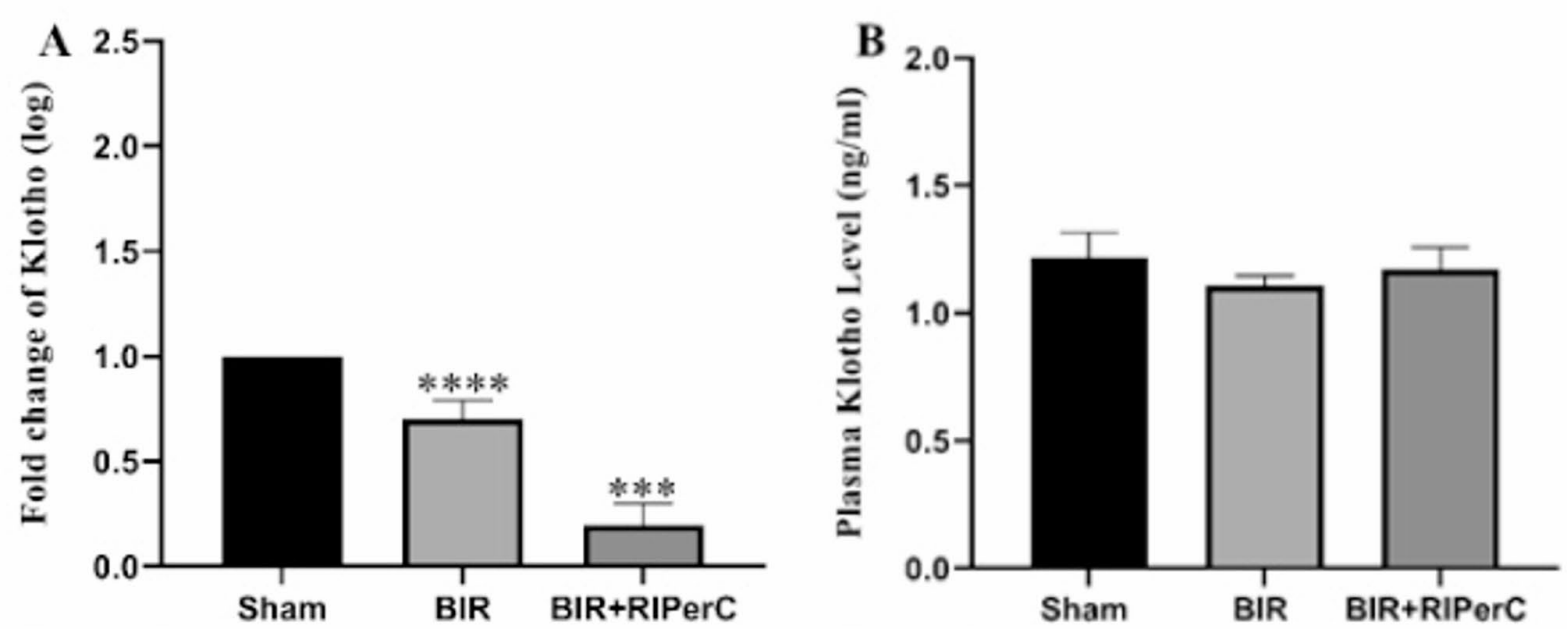
The effect of remote ischemic per-conditioning on the expression of Klotho (**A**), and plasma klotho level (**B**) after 24 h of reperfusion. Statistical analysis was performed using one-way ANOVA, followed by Tukey’s post-test. Data expressed as the mean ± SEM. ***p < 0.001 and ****p < 0.0001.RIPerC; remote ischemic per-conditioning, BIR; bilateral ischemic reperfusion

The mRNA and plasma level of Klotho decreased after 24 h of renal ischemia in the BIR group compared to the sham group. Interestingly, remote ischemic per-conditioning could not change the plasma Klotho level when BIR groups compared to the BIR + RIPerC. While, gene expression of klotho decresed in the BIR + RIPerC group compare with sham and BIR group (Fig. 6A and B).

## Discussion

In this study, we demonstrated for the first time that cyclic limb ischemic per-conditioning could not upregulate klotho expression, which downregulated during the renal ischemic reperfusion in the renal tissue. Based on animal and clinical studies, the klotho protein was subsequently reported to have a renoprotective effect and used as a therapeutic agent for renal injury [25, 26]. In the same line with Hu et al. [18], our data showed that gene expression of the klotho was markedly downregulated in the renal tissue after renal ischemia-reperfusion. Notably, the mRNA level of klotho did not rise in the rats subjected to renal I/R along with cyclic remote ischemic per conditioning. However, RIPerC corrected the functional parameters (BUN and Cr) and structural kidney damage after renal I/R injury. Our data are consistent with previous findings [27, 28] that show the hind limb per conditioning recovered the functional and structural (tubular and glomerular damages and vascular congestion) disturbance induced by renal I/R.

The kidney is susceptible to changes in oxygen concentration within its complex structure, making it sensitive to hypoxic injury when the renal pedicles are temporarily clamped. Inflammation and free radical generation are triggered during ischemia and accelerate during reperfusion [29, 30].

Participation of proinflammatory cytokines and cytokines such as TNF-α, IL-β, and IL-6 in renal ischemic reperfusion injury is strongly confirmed by the fact that pre- or post-treatments with anti-inflammatory agents have a beneficial effect in an animal renal ischemia model [31]. Hypoxia rapidly stimulates the NF-κB system in the ischemic kidney and changes the production of inflammatory and anti-inflammatory mediators [32, 33]. Our data showed a marked upregulation of NF-Κβ as well as TNF-α and IL-6 and a downregulation of IL-10 after 24 h of reperfusion in the renal tissue sample. Activation of NF-Κβ proposes that this could be an essential proximal hint for the tubular epithelial cells (TECs) to release inflammatory factors. On the other hand, Castellano et al. demonstrated a complement system involved in down-regulating the Klotho gene in renal I/RI. However, klotho expression was preserved when the activation of NF-kB signaling abrogated with complement inhibition by C1-inhibitor [34]. Our results indicated that in the BIR group, reduced Klotho expression along with decreased levels of inflammatory factors, including NF-κB. Other studies have also highlighted Klotho as a key regulator of the expression and secretion of inflammatory factors [35]. Furthermore, one study found that exogenous supplementation of Klotho or inhibition of miR-199a-5p suppressed the activity of the TLR4/NF-κB p65 signaling pathway. Thus, Klotho’s inhibitory effect on the TLR/NF-κB pathway has a crucial role in protecting organs from inflammation-induced damage and slowing the fibrotic step of the disorders [36].

In the normal condition, endogenous antioxidant enzymes (glutathione reductase, glutathione peroxidase, catalase, and superoxide dismutase) present high activity in the renal cells [37]. However, during renal ischemia and the reperfusion period, the injured cells produce excessive free radicals, causing stress. In our laboratory data, TAC level decreased, and TOS levels and OSI index were significantly increased after I/R injury. Most studies have indicated the protective effects of free radical scavengers and antioxidant agents on renal I/RI [38, 39].

Remote ischemic conditioning is an observed phenomenon that increases the tissue resistance to I/RI by a short-time cyclic of ischemia and following reperfusion before, during, or after prolonged ischemia of a distant organ [40]. Recently, studies have demonstrated the protective effect of RIPerC against renal ischemic/reperfusion injury applied via suppression of oxidative stress and downregulation of proinflammatory markers [7, 27].

In line with our previous study [26], we observed that renal epithelial cells expressed low levels of NF-Kβ, TNF-α, and IL-6 when cyclic hind limb ischemia was performed during bilateral ischemia (BIR + RIPerC group). In contrast, renal expression of anti-inflammatory cytokine (IL-10) increased in this group. In addition, RIPerC improved the oxidative stress.

On the other hand, clinical and animal studies reported that klotho protein, as an antiaging protein, identified as a novel biomarker in the diagnostic and therapeutic in AKI [17, 41]. On the basis of human study of klotho expression in AKI patient, urinary Klotho levels in patients with AKI were lower than those of healthy human controls [18]. There was a significant negative correlation between the renal Klotho score and plasma creatuinine level in the AKI patients [42]. Xie et al. [43] directly transferred the klotho gene into the bone marrow mesenchymal stem cells and injected it into rat kidneys of the renal I/R model. They found that while the expression of klotho protein upregulated, the function and pathology of kidney tissue and kidney oxidative metabolism improved after renal ischemia. RIPerC, independent of the Klotho pathway, improved inflammation and oxidative stress in the post-ischemic kidney. Therefore, these data support previous clinical and basic [44] science findings that RIPerC could be a standalone clinical intervention in cases of prolonged ischemia. Rosenberg et al. reported that ischemic conditioning in a remote organ can activate humoral factors, such as bradykinin, adenosine, and opioids. These mediators, under local innervation, induce the activation of neural pathways that lead to renal protection. Additionally, these mediators activate critical cellular pathways, including RISK (Reperfusion Injury Salvage Kinase) and SAFE (Survivor Activating Factor Enhancement), which encourage cell survival and increase tissue antioxidant defense in organs subjected to ischemia [45]. These data were recorded after 24 h of reperfusion, and this time was probably insufficient to induce Klotho expression. Therefore, reperfusion time and cyclic duration of remote ischemic perconditionong are factors that may cause we to find this results from current study. Therefore, future research with 48–72 h in the post ischemic kidney should incorporate these measurements to validate the mechanisms of action and enhance our understanding of the protective effects of treatments involving Klotho expression.

Our study has several limitations. These include the use of a single sex/species model, controlled laboratory conditions with a fixed reperfusion time (24 h), and the potential value of measuring Klotho protein levels by Western blotting or immunohistochemistry to elucidate its dynamic regulation. While rodent models provide mechanistic insights, translating these findings to human AKI pathophysiology. In future work, we will evaluate ischemic conditioning and its effects on Klotho dynamics in both male and female subjects.

## Conclusion

**Diagram 1:**
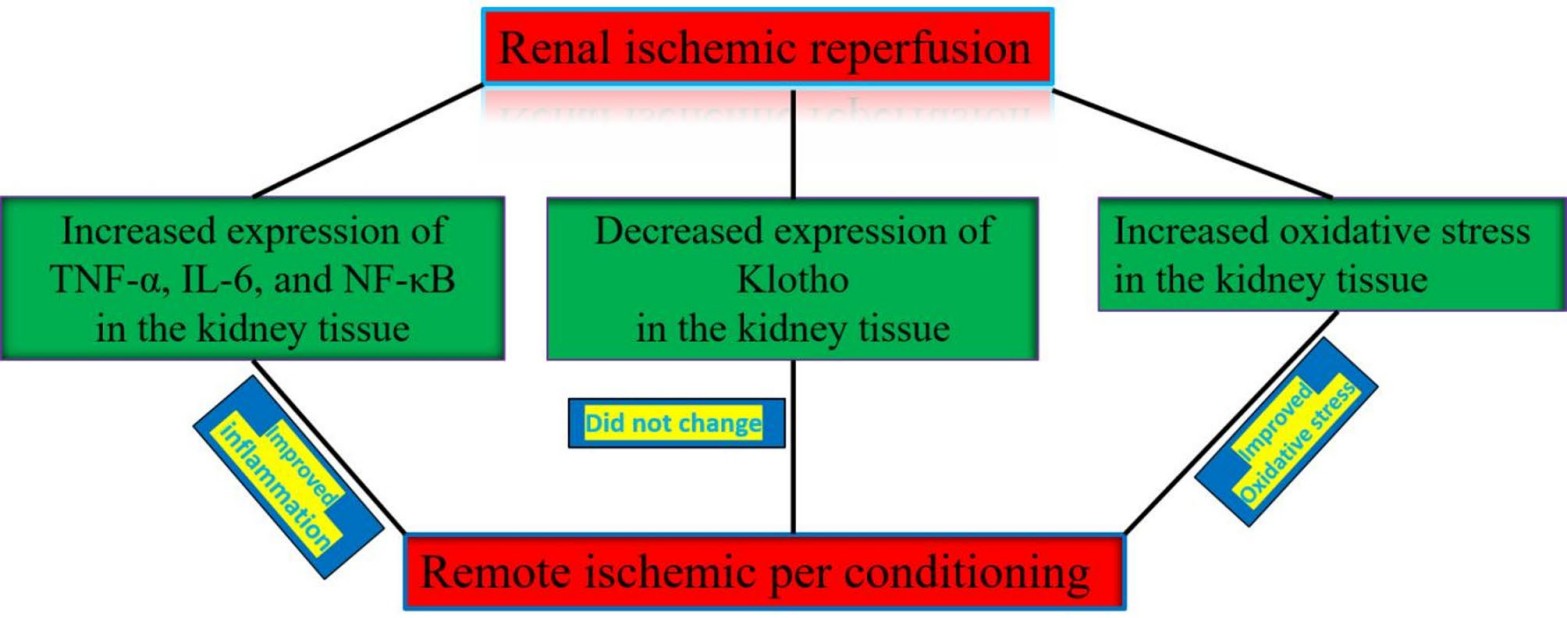
Signaling pathway

The expression of TNF-α, NF-κβ, and IL-6 as well as total oxidative stress was significantly increased after 24 h reperfusion. However, Klotho expression significantly decreased after reperfusion time. Remote ischemic per conditioning reversed inflammation and oxidative stress statues after renal ischemia, and could not upregulate klotho expression in the renal tissue. Therefore, anti-inflammatory and anti-oxidative effects of klotho do not involve the renoprotective effect of remote ischemic per conditioning against bilateral renal ischemic reperfusion injury (Diagram 1).

## Data Availability

The datasets generated and/or analyzed during the current study are not publicly available due to the need to keep them confidential but are available from the corresponding author on request.

## Abbreviations

I/RI: Renal ischemia-reperfusion injury
RIPerC: cyclic remote ischemic perconditioning
BIR: bilateral ischemic reperfusion
TAC: total antioxidative capacity
TOS: total oxidative statuse
